# Causal association between dietary factors and Primary Biliary Cholangitis: A Mendelian randomization study

**DOI:** 10.1097/MD.0000000000044873

**Published:** 2025-10-03

**Authors:** Hengli Luo, Long Han, Kai He, Kun Chen, Qiming Wei, Chengbin Zhang, Longyang Jiang

**Affiliations:** a Department of pharmacy, The Affiliated Hospital of Southwest Medical University, Luzhou, China; b Department of Pharmacy, Qingdao University Affiliated Women and Children’s Hospital, Qingdao, China; c Department of Pharmacy, Peking University People’s Hospital Qingdao Hospital, Qingdao, China; d Department of Hepatobiliary Surgery, The Affiliated Hospital of Southwest Medical University, Luzhou, China.

**Keywords:** causal association, dietary intake, genome-wide association study (GWAS), Mendelian randomization, primary biliary cholangitis (PBC)

## Abstract

The potential relationship between dietary intake and primary biliary cholangitis (PBC) remains uncertain. This study employed a 2-sample Mendelian randomization (MR) approach to investigate the causal link between dietary habits and PBC development, while also assessing potential mediating roles of PBC risk factors. We performed a 2-sample MR analysis using publicly accessible genome-wide association study data. Various MR methods including inverse-variance weighted, MR-Egger, weighted median, and others were utilized. Sensitivity analyses were conducted to ensure robustness of the findings. Following multiple testing adjustment, 2 dietary factors showed significant causal relationships with PBC. Cereal consumption was inversely associated with PBC risk (OR = 0.006, 95% CI: 0–0.089, *P* = .000198, Bonferroni-adjusted *P* = .007), whereas nonoily fish intake was positively associated (OR = 578.684, 95% CI: 24.173–13853.312, *P* = 8.64 × 10^−05^; Bonferroni-adjusted *P* = .003). Several other associations were nominally significant (unadjusted *P* < .05), including weekly beer and cider intake (Neale Lab: OR = 0.038, 95% CI: 0.002–0.597, *P* = .020; MRC-IEU: OR = 0.059, 95% CI: 0.006–0.549, *P* = .013), bread intake (OR = 61.404, 95% CI: 1.558–2419.859, *P* = .028), salad/raw vegetable intake (OR = 0.012, 95% CI: 0–0.714, *P* = .034), and tea intake (OR = 0.169, 95% CI: 0.037–0.767, *P* = .021). Notably, the effect estimates for bread and nonoily fish intake were highly imprecise, as indicated by extremely wide confidence intervals. Sensitivity analyses supported the robustness of the primary inverse-variance weighted estimates. No evidence of causal effects was found for the other dietary exposures examined. This MR study provides genetic evidence that a predisposition toward higher cereal intake may lower the risk of PBC, while a genetic tendency for nonoily fish consumption may markedly increase risk. The nominal associations observed for beer/cider, bread, salad/raw vegetables, and tea merit further investigation in larger cohorts. The implausible effect sizes for certain exposures call for cautious interpretation and additional validation.

## 1. Introduction

Primary biliary cholangitis (PBC) is a chronic, immune-mediated cholestatic liver disease characterized by progressive destruction of small intrahepatic bile ducts, leading to liver fibrosis and potentially cirrhosis.^[1]^ Globally, PBC incidence and prevalence rates range from 0.23 to 5.31 per 100,000 and 1.91 to 40.2 per 100,000, respectively.^[2]^ While North America and Nordic countries show the highest incidence, PBC prevalence is notable in Asia, particularly in China, where it ranks second highest in the Asia-Pacific region.^[3,4]^

Research into PBC pathogenesis has predominantly focused on autoimmune, genetic, and environmental factors. However, the role of dietary factors remains understudied despite their potential impact on immune function, intestinal microbiota balance, and direct liver damage.^[5]^ Understanding the relationship between dietary components and PBC not only enhances our pathogenic insights. Mendelian randomization (MR) is a genetic epidemiology method that employs germline genetic variants as instrumental variables (IVs) to assess causal relationships between exposures and outcomes. This approach mitigates confounding and reverse causation biases due to the random allocation of genetic variants at conception. In this study, we utilized MR to investigate the potential association between 6 different dietary intakes and the risk of PBC using summary data from recent genome-wide association studies.

## 2. Materials and methods

### 2.1. Study design

The study employed a 2-sample MR design to investigate causal relationships^.[6–8]^ Single nucleotide polymorphisms (SNPs) selected as IVs must satisfy 3 core assumptions: strong association with dietary exposure^[9]^; no independent influence on the occurrence of PBC.^[10]^

### 2.2. Data source

Data on dietary factors (beef intake, bread intake, cereal intake, nonoily fish intake, salad/raw vegetable intake, and tea intake) were sourced from the Neale Lab and MRC-IEU GWAS datasets. The PBC GWAS summary data included genotype information from 2861 PBC patients and 8514 controls. The specifics of the exposure and the associated outcomes are listed in (Table 1) and accessible at https://gwas.mrcieu.ac.uk/.

**Table 1:** Detailed information about the aggregated GWAS results.

GWAS ID	Trait	Consortium	Author	Sample size	SNPs (n)	Population
ukb-a-28	Average weekly beer plus cider intake	Neale Lab	Neale	241,447	10,894,596	European
ukb-b-5174	Average weekly beer plus cider intake	MRC-IEU	Ben Elsworth	327,634	9851,867	European
ukb-b-11348	Bread intake	MRC-IEU	Ben Elsworth	452,236	9851,867	European
ukb-b-15926	Cereal intake	MRC-IEU	Ben Elsworth	441,640	9851,867	European
ukb-b-17627	Nonoily fish intake	MRC-IEU	Ben Elsworth	460,880	9851,867	European
ukb-b-1996	Salad/raw vegetable intake	MRC-IEU	Ben Elsworth	435,435	9851,867	European
ukb-b-6066	Tea intake	MRC-IEU	Ben Elsworth	447,485	9851,867	European
ebi-a-GCST005581	Primary biliary cirrhosis	NA	Liu JZ	11,375	119,756	European

GWAS = genome-wide association study, SNPs = single nucleotide polymorphisms.

### 2.3. Selection of IVs

Initially, SNPs strongly associated with exposure (*P* < 5 × 10^−8^) were identified. We applied a threshold (*r*^2^ < 0.001, kb = 10,000) to select SNPs independent of linkage disequilibrium.^[9]^ SNPs absent in the outcome GWAS dataset or introducing bias due to palindromic sequences were excluded. SNPs significantly associated with the outcome (*P* < 5 × 10^−8^) were also omitted to meet instrumental variable criteria. The *F*-statistic assessed IV strength, with a threshold (*F* >10) used to avoid weak instrument bias.^[11,12]^

### 2.4. Statistical analysis

We performed a MR analysis using several methods. Our primary approach was the inverse-variance weighted (IVW) method.^[12]^ We also employed additional methods for validation, including MR-Egger regression, weighted median, simple mode, and weighted mode.^[13,14]^

Cochran *Q* statistics were used to assess heterogeneity. In cases of significant heterogeneity, we applied the multiplicative random-effects IVW model to the summary data estimates. To assess pleiotropy, we conducted the MR-Egger intercept test, where a nonzero intercept suggests the presence of horizontal pleiotropy.^[15]^ Furthermore, we utilized the MR-PRESSO global test to identify and potentially remove outlier variants, followed by repeating the analysis if necessary.^[16]^

**Figure 1.**
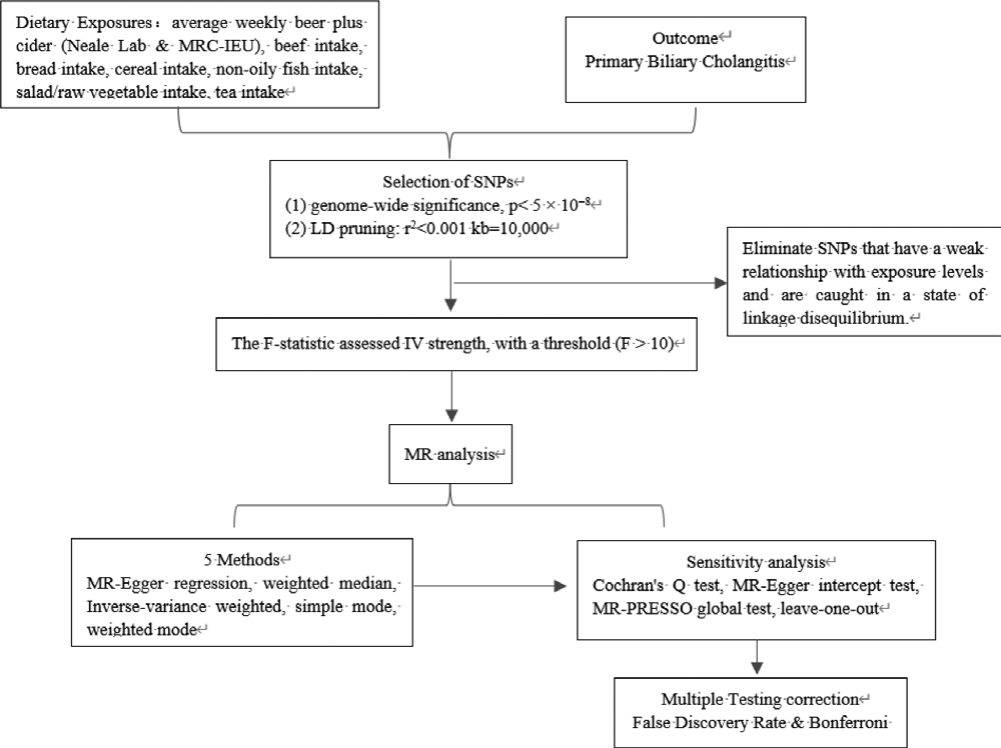
Flow chart of the MR study design. A 2-sample MR analysis was performed to examine the causal effects of dietary intake on PBC using GWAS summary statistics and the reliability of the results was tested. GWAS = genome-wide association study, IVW = inverse-variance weighted, LD = linkage disequilibrium, MR = Mendelian randomization, MR-PRESSO = Mendelian randomization pleiotropy residual sum and outlier, PBC = primary biliary cholangitis, SNPs = single nucleotide polymorphisms.

To account for multiple testing across the examined dietary exposures, the false discovery rate and Bonferroni corrections were applied.^[17]^ To evaluate the robustness of our findings, we employed the leave-one-out method.^[18]^ Detailed steps of the MR analysis are provided in Figure 1.

All analyses were conducted using R (version 4.3.0) with the Two-Sample MR and MR-PRESSO packages.^[18]^ Ethical approval and consent were not required as our study utilized existing published and public datasets.

## 3. Results

### 3.1. SNPs and instrumental variable strength

In the Methods section, MR analysis was conducted using 6 different dietary exposure factors. The exposure factor of average weekly beer plus cider intake has been studied by 2 laboratories, specifically the Neale Lab and the MRC-IEU. Consequently, 7 independent SNPs with significant p-values (*P* < 5 × 10^−8^) were selected. For average weekly beer plus cider in (Neale Lab),average weekly beer plus cider (MRC-IEU), bread, cereal, nonoily fish, salad/raw vegetables and tea, 6, 5, 4, 7, 4, 3, and 7 SNPs were selected, respectively. However, due to palindromic alleles, SNP rs9969466 (average weekly beer plus cider, MRC-IEU), rs3859193 (cereal intake), rs9427220 (salad/raw vegetables intake), and rs11164870, rs132904, rs2783129 (tea intake) were excluded. Detailed SNP information is provided in Table 2. All selected IVs had F-statistics >30, indicating minimal weak instrument bias.

**Table 2:** Summary genetic instruments between dietary habit and primary biliary cirrhosis.

SNP	Chr	EA	OA	Average weekly beer plus cider intake				Primary biliary cirrhosis				*R*_2_	*F*
				beta	eaf	se	*P*-val	beta	eaf	se	*P*-val		
rs10282264	7	T	C	0.0125317	0.477774	0.00200346	3.98 × 10^−10^	0.0285875	NA	0.0301975	.3438	7.84 × 10^−05^	39.12538529
rs10822159	10	T	C	−0.0122027	0.416902	0.00203159	1.90 × 10^−09^	0.0506931	NA	0.0305623	.0971807	7.24 × 10^−05^	36.07777418
rs1229984	4	C	T	0.0514046	0.977549	0.00716115	7.08 × 10^−13^	−0.147558	NA	0.0968966	.1278	0.000115987	51.52742345
rs34281672	8	T	C	0.0113788	0.382332	0.00207204	3.99 × 10^−08^	−0.10303	NA	0.0316138	.001118	6.12 × 10^−05^	30.15759177
rs9858059	3	T	C	−0.0123936	0.549901	0.00201407	7.59 × 10^−10^	0.0280909	NA	0.0309579	.3642	7.60 × 10^−05^	37.86568602
rs9928094	16	G	A	−0.0128795	0.422804	0.00202905	2.19 × 10^−10^	0.0676586	NA	0.0309556	.0288403	8.10 × 10^−05^	40.29141398

SNP	Chr	EA	OA	Average weekly beer plus cider intake				Primary biliary cirrhosis				R2	*F*
				beta	eaf	se	*P*-value	beta	eaf	se	*P*-value		
rs10822159	10	T	C	−0.0119112	0.417863	0.00173385	6.40 × 10^−12^	0.0506931	NA	0.0305623	.0971807	6.90 × 10^−05^	47.19413041
rs1229984	4	C	T	0.0482163	0.976527	0.00558483	6.00 × 10^−18^	−0.147558	NA	0.0968966	.1278	.000106579	74.53630257
rs1421085	16	C	T	−0.0154588	0.401556	0.00174549	8.30 × 10^−19^	0.0610951	NA	0.0311129	.0495701	.000114855	78.43625086
rs2533273	7	A	C	0.0110845	0.485739	0.00171533	1.00 × 10^−10^	0.0285875	NA	0.0302225	.3442	6.14 × 10^−05^	41.75772684
rs2883059	3	C	T	0.0116948	0.425071	0.00172733	1.30 × 10^−11^	−0.0480355	NA	0.0310871	.1223	6.68 × 10^−05^	45.83898224
rs9969466	8	C	G	0.0103307	0.566469	0.00173331	2.50 × 10^−09^	−0.0392207	NA	0.0310612	.2067	5.24 × 10^−05^	35.52278561

SNP	Chr	EA	OA	Bread intake				Primary biliary cirrhosis				R2	*F*
				beta	eaf	se	*P*-val	beta	eaf	se	*P*-val		
rs11060853	12	G	A	−0.012789	0.411804	0.00202087	2.50 × 10^−10^	−0.0270629	NA	0.0310219	.383	7.92 × 10^−05^	40.04943754
rs4665972	2	C	T	−0.0142361	0.604541	0.00204335	3.20 × 10^−12^	−0.0870947	NA	0.0313459	.00546097	9.69 × 10^−05^	48.53963829
rs6754311	2	C	T	0.0141353	0.264485	0.00224657	3.10 × 10^−10^	0.109751	NA	0.0342988	.00137499	7.77 × 10^−05^	39.58860057
rs73802707	4	T	C	−0.0159296	0.153694	0.00276165	.000000008	0.0139029	NA	0.0427815	.7452	6.60 × 10^−05^	33.2715066

SNP	Chr	EA	OA	Cereal intake				Primary biliary cirrhosis				R2	*F*
				beta	eaf	se	*P*-val	beta	eaf	se	*P*-val		
rs2450126	11	G	A	−0.0149019	0.156746	0.00245669	1.30 × 10^−09^	−0.0198966	NA	0.0422257	.6375	5.87 × 10^−05^	36.79447185
rs2504706	6	C	T	0.0181793	0.234682	0.00210238	5.30 × 10^−18^	−0.0473013	NA	0.0380485	.2138	.000118715	74.77077467
rs3115230	4	A	C	−0.0114779	0.752001	0.00207131	3.00 × 10^−08^	0.0702079	NA	0.0357348	.0494504	4.91 × 10^−05^	30.70680772
rs3859193	17	A	T	−0.0103258	0.470074	0.00179925	9.50 × 10^−09^	−0.127833	NA	0.0307967	.00003312	5.31 × 10^−05^	32.93551018
rs4988235	2	A	G	0.0114	0.736893	0.00201241	.000000015	−0.107957	NA	0.0339156	.001457	5.04 × 10^−05^	32.09052108
rs56131196	19	A	G	0.0180024	0.188724	0.00227695	2.70 × 10^−15^	−0.12897	NA	0.0393233	.00103901	9.92 × 10^−05^	62.51062288
rs62442924	7	T	C	0.0127309	0.194276	0.00225709	.000000017	−0.0458345	NA	0.0393497	.2441	5.07 × 10^−05^	31.81415996
rs9846396	3	T	C	0.0119674	0.441556	0.00180031	3.00 × 10^−11^	−0.101258	NA	0.030942	.00106601	7.06 × 10^−05^	44.18806933

SNP	Chr	EA	OA	nonoily fish intake				Primary biliary cirrhosis				R2	*F*
				beta	eaf	se	*P*-val	beta	eaf	se	*P*-val		
rs1260326	2	C	T	−0.00955354	0.604249	0.0016553	7.90 × 10^−09^	−0.0880109	NA	0.0312218	.00481903	4.37 × 10^−05^	33.31004524
rs16822430	2	C	T	0.0116344	0.23321	0.00191996	1.40 × 10^−09^	0.0227395	NA	0.0354901	.5217	4.84 × 10^−05^	36.72008016
rs4318925	6	T	C	−0.0150377	0.177245	0.0021207	1.30 × 10^−12^	−0.134675	NA	0.0393555	.000621599	6.60 × 10^−05^	50.28105253
rs56094641	16	G	A	0.0125875	0.404619	0.00165108	2.50 × 10^−14^	0.0667236	NA	0.0312162	.0325597	7.63 × 10^−05^	58.12229223

SNP	Chr	EA	OA	Salad/raw vegetable intake				Primary biliary cirrhosis				R2	F
				beta	eaf	se	*P*-val	beta	eaf	se	*P*-val		
rs1052352	16	T	C	0.00816444	0.52355	0.00142564	.00000001	−0.0459289	NA	0.0305551	.1328	3.33 × 10^−05^	32.79691991
rs10819082	9	A	G	−0.00916475	0.66734	0.00151342	1.40 × 10^−09^	0.0036065	NA	0.0332786	.9137	3.73 × 10^−05^	36.67096236
rs12203592	6	T	C	−0.0102862	0.219338	0.00169411	1.30 × 10^−09^	0.0732505	NA	0.0359106	.0413704	3.62 × 10^−05^	36.86605923
rs9427220	1	T	A	−0.00800801	0.554661	0.00144212	2.80 × 10^−0^8	−0.00299551	NA	0.0296399	.9195	3.17 × 10^−05^	30.83517474

SNP	Chr	EA	OA	Tea intake				Primary biliary cirrhosis				R2	F
				beta	eaf	se	*P*-val	beta	eaf	se	*P*-val		
rs11164870	1	G	C	−0.0119604	0.604574	0.00218232	4.20 × 10^−08^	0.0134906	NA	0.0319138	.6725	6.84 × 10^−05^	30.03686088
rs1156588	2	G	A	−0.015454	0.210071	0.00260325	2.90 × 10^−09^	0.0324672	NA	0.0390223	.4054	7.93 × 10^−05^	35.24114916
rs132904	22	C	G	0.0166007	0.778651	0.00255257	7.80 × 10^−11^	−0.123102	NA	0.0370815	.000900907	9.50 × 10^−05^	42.29582311
rs1481012	4	G	A	−0.0262435	0.112209	0.00335608	5.30 × 10^−15^	−0.0147076	NA	0.0473714	.756201	.000137218	61.14752739
rs2279844	17	A	G	−0.0119879	0.379343	0.00218318	.00000004	0.0516432	NA	0.0315631	.1018	6.77 × 10^−05^	30.15137588
rs2478875	6	G	A	0.0218943	0.208758	0.00261129	5.10 × 10^−17^	−0.0288111	NA	0.0398781	.47	.00015836	70.29944849
rs2783129	13	G	C	−0.0117331	0.484878	0.00213314	3.80 × 10^−08^	−0.0198026	NA	0.0310032	.523	6.88 × 10^−05^	30.25428023
rs57462170	3	A	G	0.0191505	0.108773	0.00340563	1.90 × 10^−08^	0.0392207	NA	0.0468893	.4029	7.11 × 10^−05^	31.62025072
rs9624470	22	A	G	0.0252071	0.580054	0.00215485	1.30 × 10^−31^	−0.0657877	NA	0.0309735	.0336698	.000309555	136.8395635
rs9937354	16	A	G	−0.0140923	0.424074	0.0021433	4.90 × 10^−11^	0.0610951	NA	0.0311129	.0495701	9.70 × 10^−05^	43.23125388

SNPs = single nucleotide polymorphisms.

### 3.2. Two-sample MR analyses results

The IVW analysis indicated several associations between dietary factors and PBC risk. After applying a strict Bonferroni correction for multiple testing (significance threshold set at *P* < .0071), cereal intake (OR = 0.006, 95% CI: 0–0.089, *P* = .000198; Bonferroni-adjusted *P* = .007) (Table 3 and Fig. 5A and D) remained statistically significant, indicating a strong protective effect, while nonoily fish intake (OR = 578.684, 95% CI: 24.173–13853.312, *P* = 8.64 × 10^−05^; Bonferroni-adjusted *P* = .003) (Table 3 and Fig. 6A and D) indicated a strong risk factor for PBC.

**Table 3:** Results of the 2-sample MR analyses.

Exposure	Outcome	Method	SNPs (n)	beta	se	*P*-val	OR (95% CI)	Bonferroni_adjusted	OR (95% CI)
Average weekly beer plus cider intake(ukb-a-28)	Primary biliary cirrhosis(ebi-a-GCST005581)	MR-Egger	6	–2.273831522	4.030764964	.602801542	0.671499503	1	0.103 (0–277.68)
		Weighted median	6	–3.09646058	1.378484624	.024686165	0.08107204	0.864015775	0.045 (0.003–0.674)
		Inverse-variance weighted	6	–3.274090681	1.407208659	.019983573	0.08107204	0.699425055	0.038 (0.002–0.597)
		Simple mode	6	–3.483005254	1.923352531	.129921276	0.194485499	1	0.031 (0.001–1.332)
		Weighted mode	6	–3.251803955	1.701314417	.11418865	0.181663761	1	0.039 (0.001–1.086)
Average weekly beer plus cider intake(ukb-b-5174)	Primary biliary cirrhosis(ebi-a-GCST005581)	MR-Egger	5	–4.049210401	3.235650819	.299473385	0.361433396	1	0.017 (0–9.901)
		Weighted median	5	–3.776200229	1.311775916	.003993268	0.027952876	0.13976438	0.023 (0.002–0.3)
		Inverse-variance weighted	5	–2.834147423	1.140510553	.012955823	0.064779115	0.453453805	0.059 (0.006–0.549)
		Simple mode	5	–4.110971278	1.69580871	.072433786	0.126759126	1	0.016 (0.001–0.455)
		Weighted mode	5	–4.077320503	1.506340405	.053716353	0.104448464	1	0.017 (0.001–0.325)
Bread intake(ukb-b-11348)	Primary biliary cirrhosis(ebi-a-GCST005581)	MR-Egger	4	−6.630255797	30.62294031	.848665675	0.848665675	1	0.001 (0–1.539E + 23)
		Weighted median	4	4.143985128	1.505996573	.00592947	0.034588575	0.20753145	63.054 (3.294–1206.813)
		Inverse-variance weighted	4	4.117473627	1.87448521	.028049811	0.081811949	0.981743385	61.404 (1.558–2419.859)
		Simple mode	4	5.927029773	3.019875992	.144474942	0.194485499	1	375.039 (1.008–139523.276)
		Weighted mode	4	5.637951494	2.868238086	.14405623	0.194485499	1	280.887 (1.016–77629.139)
Cereal intake(ukb-b-15926)	Primary biliary cirrhosis(ebi-a-GCST005581)	MR-Egger	7	2.216537268	6.996741202	.764198533	0.798342823	1	9.176 (0–8286597.626)
		Weighted median	7	−5.29436211	1.406686211	.00016741	0.002315122	0.00585935	0.005 (0–0.079)
		Inverse-variance weighted	7	−5.105111996	1.371974682	.000198439	0.002315122	0.006945365	0.006 (0–0.089)
		Simple mode	7	−7.058714169	2.57193297	.033534372	0.084737452	1	0.001 (0–0.133)
		Weighted mode	7	−6.627960627	2.543202267	.040328246	0.092188058	1	0.001 (0–0.193)
nonoily fish intake(ukb-b-17627)	Primary biliary cirrhosis (ebi-a-GCST005581)	MR-Egger	4	7.549957913	12.62091599	.610421401	0.671499503	1	1900.663 (0–1.052E + 14)
		Weighted median	4	6.492170118	1.79168832	.000290651	0.002543196	0.010172785	659.954 (19.697–22112.077)
		Inverse-variance weighted	4	6.360756282	1.620164981	8.63727 × 10^−05^	0.002315122	0.003023045	578.684 (24.173–13853.312)
		Simple mode	4	8.67107672	3.071669711	.066577592	0.122642933	1	5831.775 (14.163–2401368.53)
		Weighted mode	4	8.147115126	2.623541049	.053071926	0.104448464	1	3453.402 (20.186–590813.442)
Salad/raw vegetable intake(ukb-b-1996)	Primary biliary cirrhosis(ebi-a-GCST005581)	MR-Egger	3	−11.07654057	30.10186857	.775533028	0.798342823	1	0 (0–6.498E + 20)
		Weighted median	3	−5.548750317	2.730544235	.042143112	0.092188058	1	0.004 (0–0.821)
		Inverse-variance weighted	3	−4.430122817	2.088381417	.033894981	0.084737452	1	0.012 (0–0.714)
		Simple mode	3	−6.363567462	3.663698016	.224533672	0.283106584	1	0.002 (0–2.264)
		Weighted mode	3	−6.427522998	3.723895023	.226485267	0.283106584	1	0.002 (0–2.39)
Tea intake(ukb-b-6066)	Primary biliary cirrhosis(ebi-a-GCST005581)	MR-Egger	7	1.424874332	2.650698012	.613942403	0.671499503	1	4.157 (0.023–750.127)
		Weighted median	7	−2.22458079	0.995761422	.025479784	0.08107204	0.89179244	0.108 (0.015–0.761)
		Inverse-variance weighted	7	−1.77596428	0.770532588	.021174812	0.08107204	0.74111842	0.169 (0.037–0.767)
		Simple mode	7	−2.635775905	1.561306874	.142341856	0.194485499	1	0.072 (0.003–1.529)
		Weighted mode	7	−2.369855016	1.241259892	.104819253	0.174698755	1	0.093 (0.008–1.065)

MR = Mendelian randomization, OR = odds ratio, SNPs = single nucleotide polymorphisms.

**Figure 2.**
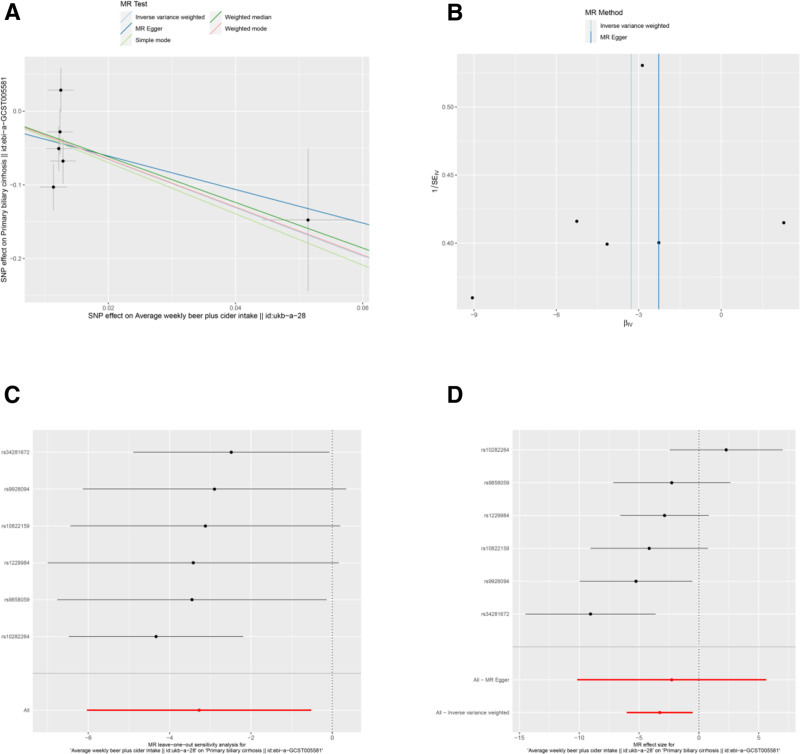
Sensitivity analyses of the relationship between average weekly beer plus cider intake (Neale Lab) and the risk of PBC. (A) scatter plot: illustrates the association between SNPs and both weekly beer and cider intake and PBC risk. (B) funnel plot: assesses the symmetry of the standard error distribution, indicating potential publication bias. (C) Leave-one-out analysis: evaluates the robustness of the PBC risk association by excluding 1 SNP at a time. (D) Forest plot: presents the pooled effects of average weekly beer plus cider intake (Neale Lab) on PBC risk, with confidence intervals for each SNP. PBC = primary biliary cholangitis, SNPs = single nucleotide polymorphisms.

**Figure 3.**
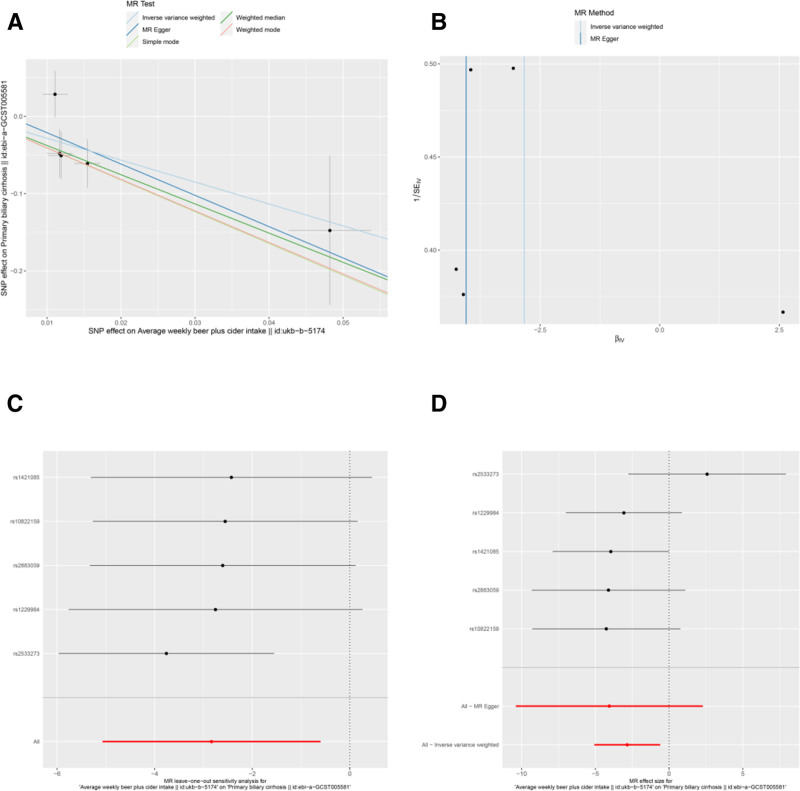
Sensitivity analyses of the relationship between average weekly beer plus cider intake (MRC-IEU) and the risk of PBC. (A) scatter plot: illustrates the association between SNPs and both weekly beer and cider intake (MRC-IEU) and PBC risk. (B) Funnel plot: assesses the symmetry of the standard error distribution, indicating potential publication bias. (C) Leave-one-out analysis: evaluates the robustness of the PBC risk association by excluding 1 SNP at a time. (D) Forest plot: presents the pooled effects of average weekly beer plus cider intake (MRC-IEU) on PBC risk, with confidence intervals for each SNP. PBC = primary biliary cholangitis, SNPs = single nucleotide polymorphisms.

**Figure 4.**
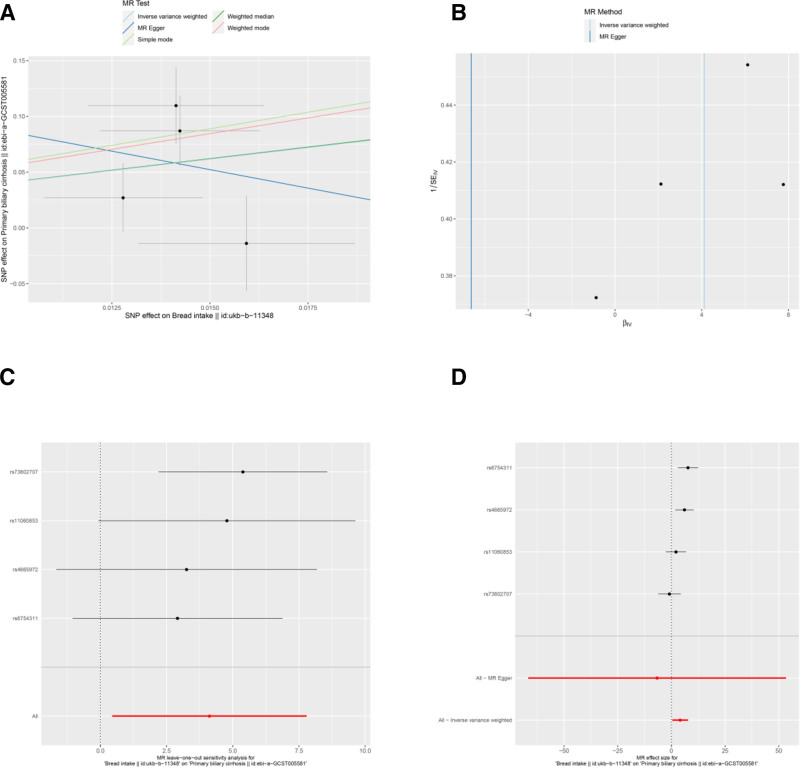
Sensitivity analyses of the relationship between bread intake and the risk of PBC. (A) Scatter plot: illustrates the association between SNPs and both bread intake and PBC risk. (B) Funnel plot: assesses the symmetry of the standard error distribution, indicating potential publication bias. (C) Leave-one-out analysis: evaluates the robustness of the PBC risk association by excluding 1 SNP at a time. (D) Forest plot: presents the pooled effects of bread intake on PBC risk, with confidence intervals for each SNP. PBC = primary biliary cholangitis, SNPs = single nucleotide polymorphisms.

Several other associations were nominally significant (uncorrected *P* < .05) but did not survive multiple testing correction. These included potential protective factors: average weekly beer plus cider intake (Neale Lab) (OR = 0.038, 95% CI: 0.002–0.597, *P* = .020) (Table 3 and Fig. 2A and D), average weekly beer plus cider intake (MRC-IEU) (OR = 0.059, 95% CI: 0.006–0.549, *P* = .013) (Table 3 and Fig. 3A and D), salad/raw vegetable intake (OR = 0.012, 95% CI: 0–0.714, *P* = .034) (Table 3 and Fig. 7A, D), and tea intake (OR = 0.169, 95% CI: 0.037–0.767, *P* = .021) (Table 3 and Fig. 8A and D). Bread intake (OR = 61.404, 95% CI: 1.558–2419.859, *P* = .028) was identified as a nominally significant risk factor (Table 3 and Fig. 4A and D). These nominally significant findings should be interpreted with caution and require replication in future studies.

**Figure 5.**
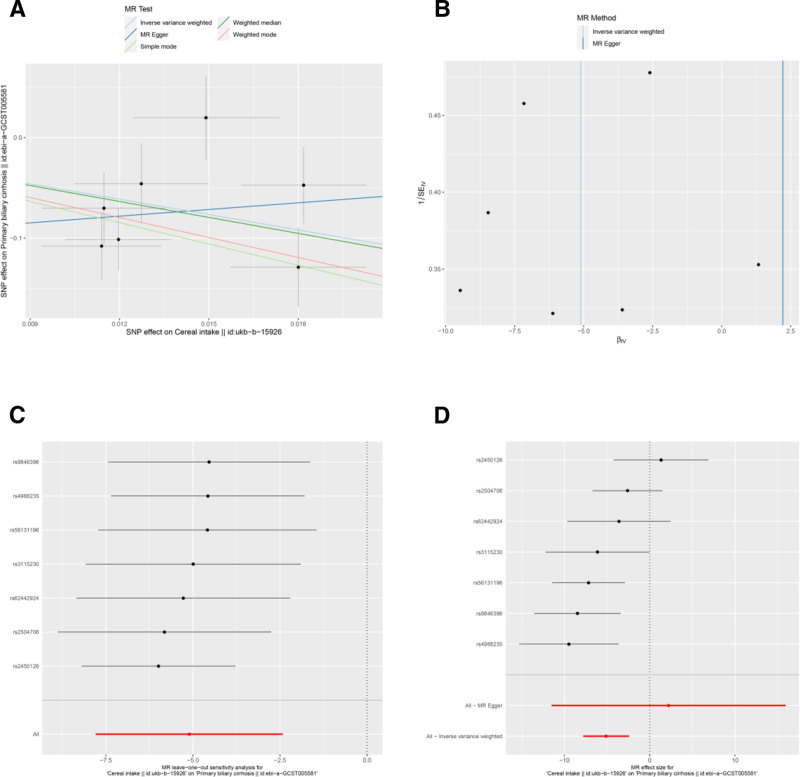
Sensitivity analyses of the relationship between cereal intake and the risk of PBC. (A) Scatter plot: illustrates the association between SNPs and cereal intake and PBC risk. (B) Funnel plot: assesses the symmetry of the standard error distribution, indicating potential publication bias. (C) Leave-one-out analysis: evaluates the robustness of the PBC risk association by excluding 1 SNP at a time. (D) Forest plot: presents the pooled effects of cereal intake on PBC risk, with confidence intervals for each SNP. PBC = primary biliary cholangitis, SNPs = single nucleotide polymorphisms.

**Figure 6.**
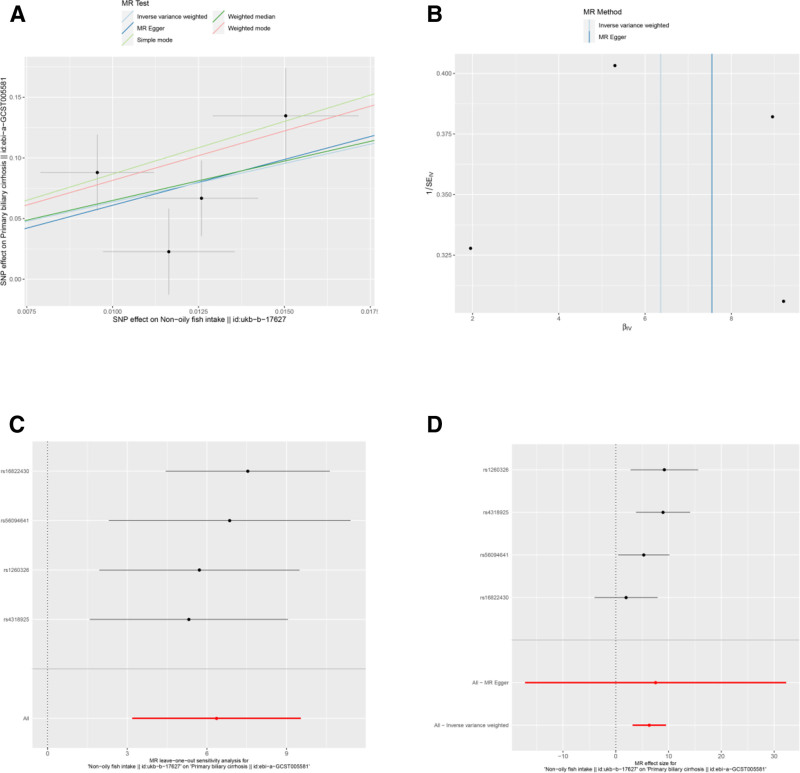
Sensitivity analyses of the relationship between nonoily fish intake and the risk of PBC. (A) Scatter plot: illustrates the association between SNPs and both nonoily fish intake and PBC risk. (B) Funnel plot: assesses the symmetry of the standard error distribution, indicating potential publication bias. (C) Leave-one-out analysis: evaluates the robustness of the PBC risk association by excluding 1 SNP at a time. (D) Forest Plot: Presents the pooled effects of nonoily fish intake on PBC risk, with confidence intervals for each SNP. PBC = primary biliary cholangitis, SNPs = single nucleotide polymorphisms.

**Figure 7.**
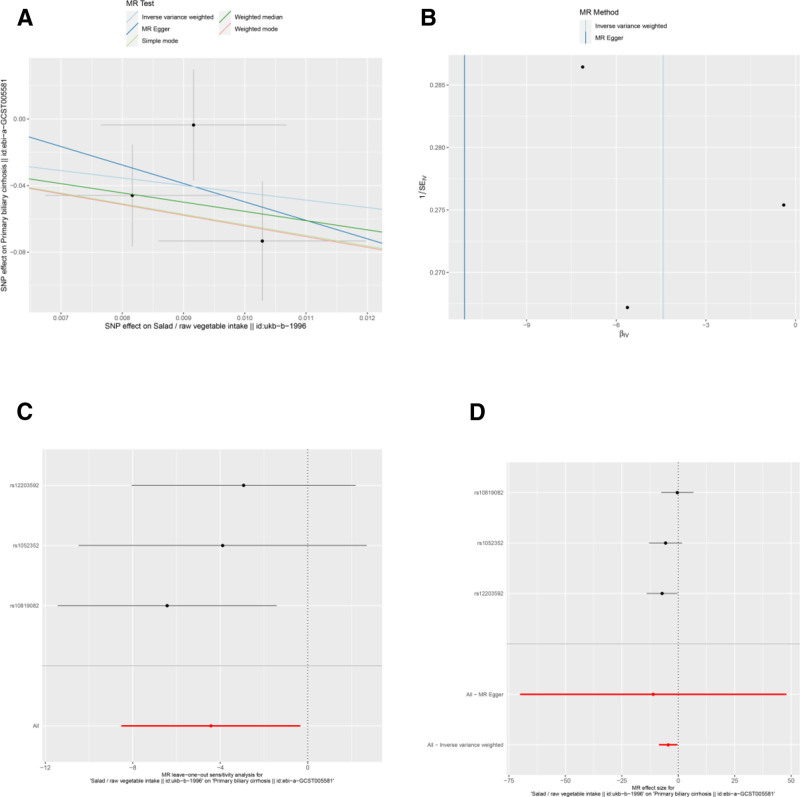
Sensitivity analyses of the relationship between salad/raw vegetable intake and the risk of PBC. (A) Scatter plot: illustrates the association between SNPs and both salad/raw vegetable intake and PBC risk. (B) Funnel plot: assesses the symmetry of the standard error distribution, indicating potential publication bias. (C) Leave-one-out analysis: evaluates the robustness of the PBC risk association by excluding 1 SNP at a time. (D) Forest plot: presents the pooled effects of salad/raw vegetable intake on PBC risk, with confidence intervals for each SNP. PBC = primary biliary cholangitis, SNPs = single nucleotide polymorphisms.

**Figure 8.**
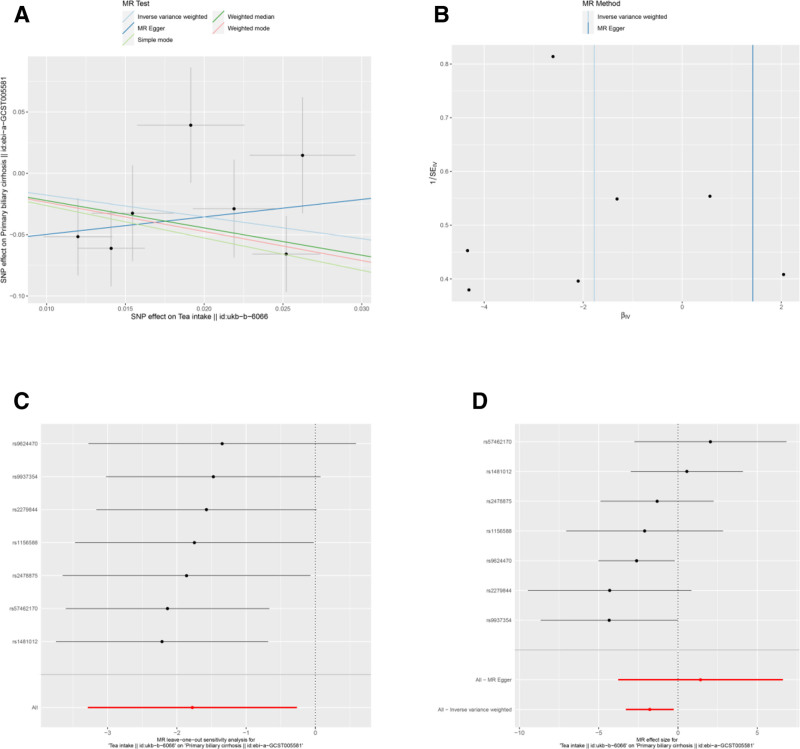
Sensitivity analyses of the relationship between tea intake and the risk of PBC. (A) Scatter plot: illustrates the association between SNPs and both tea intake and PBC risk. (B) Funnel plot: assesses the symmetry of the standard error distribution, indicating potential publication bias. (C) Leave-one-out analysis: evaluates the robustness of the PBC risk association by excluding 1 SNP at a time. (D) Forest plot: presents the pooled effects of tea intake on PBC risk, with confidence intervals for each SNP. PBC = primary biliary cholangitis, SNPs = single nucleotide polymorphisms.

### 3.3. Evaluation of reliability

Results from the MR-Egger intercept, Cochran *Q* heterogeneity, and MR-PRESSO global tests (Table 4) were statistically insignificant(all *P* > .05), indicating the reliability of the MR analysis. funnel plots displayed symmetrical distributions suggesting that no single SNP exerted disproportionate influence on the outcomes (Figs 2B, 3B, 4B, 5B, 6B, 7B, and 8B). Moreover, The leave-one-out method showed consistent results with *P*-values >.05 after sequentially removing each SNP (Figs. 2C, 3C, 4C, 5C, 6C, 7C, and 8C).

**Table 4:** Reliability test of MR analysis results.

Exposure	Outcome	Method	Q	Q_df	Q_*P*val	MR-Egger intercept test *P*	MR-PRESSO global test *P*
Primary biliary cirrhosis ‖ id:ebi-a-GCST005581	Average weekly beer plus cider intake ‖ id:ukb-a-28	MR-Egger	10.46428686	4	0.033292501	–	–
		IVW	10.65373879	5	0.058694368	.80115815	.103
Primary biliary cirrhosis ‖ id:ebi-a-GCST005581	Average weekly beer plus cider intake ‖ id:ukb-b-5174	MR-Egger	4.545916713	3	0.208231007	–	–
		IVW	4.799371595	4	0.308509457	.710000211	.51
Primary biliary cirrhosis ‖ id:ebi-a-GCST005581	Bread intake ‖ id:ukb-b-11348	MR-Egger	6.796710742	2	0.033428202	–	–
		IVW	7.217546975	3	0.065277749	.758530795	.147
Primary biliary cirrhosis ‖ id:ebi-a-GCST005581	Cereal intake ‖ id:ukb-b-15926	MR-Egger	9.502324565	5	0.090629113	–	–
		IVW	11.66466794	6	0.06988162	.334891623	.331
Primary biliary cirrhosis ‖ id:ebi-a-GCST005581	nonoily fish intake ‖ id:ukb-b-17627	MR-Egger	3.995565358	2	0.135635698	–	–
		IVW	4.013749708	3	0.259983233	.932690906	.401
Primary biliary cirrhosis ‖ id:ebi-a-GCST005581	Salad/raw vegetable intake ‖ id:ukb-b-1996	MR-Egger	1.841426668	1	0.174783825	–	–
		IVW	1.932001838	2	0.380602057	.861057785	.426
Primary biliary cirrhosis ‖ id:ebi-a-GCST005581	Tea intake ‖ id:ukb-b-6066	MR-Egger	5.26158394	5	0.384797255	–	–
		IVW	6.924143046	6	0.327919458	.264294353	.14

IVW = inverse-variance weighted, MR = Mendelian randomization, MR-PRESSO = Mendelian randomization pleiotropy residual sum and outlier, SNPs = single nucleotide polymorphisms.

## 4. Discussion

PBC is an autoimmune liver disease characterized by progressive bile duct destruction, leading to fibrosis and cirrhosis. While genetic predisposition plays a significant role in PBC pathogenesis,^[19,20]^ environmental factors, including dietary habits and their interaction with the gut microbiota, are increasingly recognized as potential triggers.^[21,22]^ Our study utilized MR to assess the causal relationship between 6 dietary factors and PBC risk using genome-wide association study (GWAS) data. After rigorous multiple testing correction, only higher cereal intake (protective) and nonoily fish intake (risk factor) demonstrated strong evidence for a causal association with PBC. We also observed several nominally significant associations (beer/cider, salad/raw vegetables, tea, and bread) which, while potentially indicative of underlying biological relationships, must be considered exploratory and warrant further validation in independent cohorts.

Average weekly beer plus cider intake: Our findings suggest a potential protective effect of higher beer and cider consumption against PBC.Interestingly, a population-based case-control study found that alcohol consumption reduces the risk of autoimmune hepatitis, indicating it might be protective against developing PBC.^[23]^The mechanisms involve interactions with the gut microbiome and metabolic pathways influenced by compounds such as polyphenols and ethanol.^[24–26]^

Polyphenols in beer and cider possess antioxidant and anti-inflammatory properties that may modulate immune responses and gut microbiota composition, potentially lowering PBC risk.^[27–30]^ Ethanol, a major component, can affect immune cell signaling and function, although its precise role in PBC requires further investigation.^[31,32]^

Cereal intake: This study found strong evidence supporting a protective causal effect of cereal intake against PBC, surviving strict multiple testing correction. Cereals, particularly whole grains, are rich in fiber and bioactive compounds that support gut health and immune function. The prebiotic effects of dietary fiber stimulate beneficial gut bacteria, which are crucial for immune homeostasis and may reduce the risk of autoimmune conditions like PBC.^[33–35]^ Additionally, antioxidants and other bioactive components in cereals contribute to anti-inflammatory mechanisms, supporting overall liver health.^[36,37]^

Salad/raw vegetable intake: Nominally significant results suggested a potential protective role. Vegetables and fruits are rich sources of antioxidants, vitamins, and minerals, which exert protective effects against inflammation and oxidative stress associated with PBC.^[38,39]^ Specific vegetables like dark-green and red-orange varieties contain polyphenols and carotenoids known for their anti-inflammatory properties, potentially mitigating PBC risk through immune modulation and antioxidant activities.^[40,41]^ The fiber content in vegetables supports a diverse gut microbiota, further enhancing immune function and reducing autoimmune responses.^[42]^

Tea intake: A nominally significant protective association was observed. Tea, particularly green tea, is abundant in polyphenols such as catechins, which possess strong antioxidant and anti-inflammatory properties^.[43–45]^ These compounds may protect against liver diseases by neutralizing free radicals and modulating immune responses, suggesting a potential benefit in lowering PBC risk.^[46]^

Nonoily fish and bread intake: In contrast, our study identified nonoily fish intake as a strong risk factor for PBC after multiple testing correction. nonoily fish provide essential nutrients but may also influence the gut microbiota and immune responses in ways that could exacerbate autoimmune conditions, though the mechanisms warrant further exploration.^[47–49]^ Bread intake was associated with an increased risk at a nominal significance level. Particularly when high in advanced glycation end-products (AGEs), could promote inflammation and oxidative stress, potentially increasing the risk of liver diseases like PBC.^[50–52]^

Our MR study employed rigorous methodologies to ensure robust causal inference. We applied strict Bonferroni correction to account for multiple testing, a step that strengthens the credibility of our findings by reducing the likelihood of Type I errors.^[17]^ While this approach confirmed the robustness of the associations for cereal and nonoily fish intake, it also highlighted that several other associations (e.g., beer/cider, bread, salad/raw vegetables, tea), though nominally significant, should be interpreted with caution as exploratory and require replication in larger studies. The extreme effect sizes (ORs) observed for some exposures, such as nonoily fish and bread intake, while potentially reflecting true biological relationships, may also be influenced by the limited number of strong genetic instruments available for complex dietary traits and the relative rarity of the PBC outcome. This is a known challenge in MR studies. To mitigate concerns regarding weak instruments, we ensured all SNPs used had F-statistics > 30, effectively minimizing this bias. Furthermore, we conducted comprehensive sensitivity analyses. The nonsignificant results from the MR-Egger intercept tests and MR-PRESSO global tests (all *P* >.05) suggest that horizontal pleiotropy is unlikely to be a major source of bias for our primary findings. Where Cochran Q test indicated heterogeneity (e.g., for some beer/cider and bread analyses), we utilized the multiplicative random-effects IVW model to provide more conservative and reliable estimates. Nonetheless, the limited number of SNPs for certain exposures inherently reduces the power of some sensitivity tests, such as MR-Egger, and contributes to wider confidence intervals. This underscores the importance of interpreting these results as generating hypotheses for future investigation rather than providing definitive conclusions.

Our study has limitations. Firstly, the GWAS data predominantly included European populations, limiting generalizability to other ethnicities. Secondly, while we assessed causal relationships for 6 dietary factors, other dietary variables were not included and require future investigation. Thirdly, the lack of age-specific data precluded stratified analyses. Lastly, despite efforts to mitigate confounding, residual biases may persist, emphasizing the need for further clinical validation.

## 5. Conclusion

Our findings indicate that cereal intake may play a protective role against PBC, while nonoily fish intake appears to be a significant risk factor, based on associations that survived rigorous multiple testing correction. Nominally significant associations suggest that weekly beer plus cider, salad/raw vegetables, and tea intake might be protective, whereas bread intake might increase risk; however, these latter findings require further replication and validation. Further exploration into the underlying mechanisms driving these associations is warranted.

## Author contributions

**Conceptualization:** Longyang Jiang.

**Data curation:** Long Han, Kun Chen.

**Formal analysis:** Qiming Wei, Chengbin Zhang.

**Writing – original draft:** Hengli Luo.

**Writing – review & editing:** Kai He, Longyang Jiang.
